# Reduced peripheral arterial blood flow with preserved cardiac output during submaximal bicycle exercise in elderly heart failure

**DOI:** 10.1186/1532-429X-11-48

**Published:** 2009-11-18

**Authors:** Chirapa Puntawangkoon, Dalane W Kitzman, Stephen B Kritchevsky, Craig A Hamilton, Barbara Nicklas, Xiaoyan Leng, Peter H Brubaker, W Gregory Hundley

**Affiliations:** 1 Department of Biomedical Engineering, Wake Forest University School of Medicine, Medical Center Boulevard, Winston-Salem, North Carolina, 27157, USA; 2 Department of Biostastical Sciences, Wake Forest University School of Medicine, Medical Center Boulevard, Winston-Salem, North Carolina, 27157, USA; 3 Department of Internal Medicine - Cardiology Section, Wake Forest University School of Medicine, Medical Center Boulevard, Winston-Salem, North Carolina, 27157, USA; 4 Department of Internal Medicine - Geriatrics & Gerontology, Wake Forest University School of Medicine, Medical Center Boulevard, Winston-Salem, North Carolina, 27157, USA; 5 Department of Human Genomics, Wake Forest University School of Medicine, Medical Center Boulevard, Winston-Salem, North Carolina, 27157, USA; 6 Department of Radiology, Wake Forest University School of Medicine, Medical Center Boulevard, Winston-Salem, North Carolina, 27157, USA; 7 Department of Public Health Sciences, Wake Forest University School of Medicine, Medical Center Boulevard, Winston-Salem, North Carolina, 27157, USA; 8 Department of Health and Exercise Science, Wake Forest University School of Medicine, Medical Center Boulevard, Winston-Salem, North Carolina, 27157, USA

## Abstract

**Background:**

Older heart failure (HF) patients exhibit exercise intolerance during activities of daily living. We hypothesized that reduced lower extremity blood flow (LBF) due to reduced forward cardiac output would contribute to submaximal exercise intolerance in older HF patients.

**Methods and Results:**

Twelve HF patients both with preserved and reduced left ventricular ejection fraction (LVEF) (aged 68 ± 10 years) without large (aorta) or medium sized (iliac or femoral artery) vessel atherosclerosis, and 13 age and gender matched healthy volunteers underwent a sophisticated battery of assessments including a) peak exercise oxygen consumption (peak VO_2_), b) physical function, c) cardiovascular magnetic resonance (CMR) submaximal exercise measures of aortic and femoral arterial blood flow, and d) determination of thigh muscle area. Peak VO_2 _was reduced in HF subjects (14 ± 3 ml/kg/min) compared to healthy elderly subjects (20 ± 6 ml/kg/min) (p = 0.01). Four-meter walk speed was 1.35 ± 0.24 m/sec in healthy elderly verses 0.98 ± 0.15 m/sec in HF subjects (p < 0.001). After submaximal exercise, the change in superficial femoral LBF was reduced in HF participants (79 ± 92 ml/min) compared to healthy elderly (222 ± 108 ml/min; p = 0.002). This occurred even though submaximal stress-induced measures of the flow in the descending aorta (5.0 ± 1.2 vs. 5.1 ± 1.3 L/min; p = 0.87), and the stress-resting baseline difference in aortic flow (1.6 ± 0.8 vs. 1.7 ± 0.8 L/min; p = 0.75) were similar between the 2 groups. Importantly, the difference in submaximal exercise induced superficial femoral LBF between the 2 groups persisted after accounting for age, gender, body surface area, LVEF, and thigh muscle area (*p *≤ 0.03).

**Conclusion:**

During CMR submaximal bike exercise in the elderly with heart failure, mechanisms other than low cardiac output are responsible for reduced lower extremity blood flow.

## Background

Heart failure (HF) patients often exhibit reduced exercise-induced lower extremity blood flow (LBF) that correlates with their severe exercise intolerance[1,2]. The failure of muscle blood flow to increase during maximal exercise in patients with HF has been shown to be mediated in part by reduced maximal exercise-induced cardiac output[2,3]. In the elderly, HF limits many activities of daily living, which are performed during submaximal as opposed to maximal exercise[4]. Understanding the relationship between cardiac output and LBF during submaximal exercise in the elderly would be useful for designing treatment strategies that could preserve the ability of older individuals to perform activities of daily living.

Accordingly, we hypothesized that reduced cardiac output would contribute to reduced superficial femoral arterial LBF during submaximal exercise. To address this hypothesis, we performed cardiovascular magnetic resonance (CMR) in elderly participants in which aorta and superficial femoral arterial blood flow was measured before and after submaximal exercise. In these same participants, we also performed assessments of middle thigh skeletal muscle area, physical function, and peak exercise capacity using expired gas analysis.

## Methods

### Study Population

The study was approved by the Institutional Review Board of the Wake Forest University School of Medicine, and all participants provided informed consent. The study population consisted of 25 individuals aged 57-82 years: 13 were healthy subjects, and 12 participants had HF. The diagnosis of HF was based on clinical criteria that included: (1) a HF clinical score from National Health and Nutrition Examination Survey-I of ≥ 3,[5] and (2) those utilized previously, including a history of acute pulmonary edema or the occurrence of at least 2 of the following that improved with diuretic therapy without another identifiable cause: dyspnea on exertion, paroxysmal nocturnal dyspnea, orthopnea, bilateral lower extremity edema, or exertional fatigue[5-7]. A range of left ventricular ejection fraction (LVEF) was recruited into the study. The healthy community-dwelling volunteers were aged ≥ 55 years, took no heart failure medications, had no chronic medical disease, had a normal physical examination, had a systolic and diastolic blood pressure below 140 and 90 mmHg, respectively, normal pulmonary function tests, and normal serum chemistries with normal rest and exercise stress echocardiogram.

Participants were ineligible for enrollment into the HF or healthy group if they had; (1) cognitive impairment; (2) alcoholic consumption > 2 drinks/day within the preceding 2 months; (3) current tobacco use; (4) an inability to exercise; (5) any historical, physical exam, or imaging evidence of peripheral or carotid vascular disease; (6) evidence of myocardial ischemia or infarction within 12 months of study participation; (7) a contraindication for CMR scanning (e.g. claustrophobia, pacemakers, defibrillators or other implanted electronic device); (8) a stroke within 6 months; (9) active treatment cancer; (10) anemia (<11 gm hemoglobin), (11) renal insufficiency defined as a serum creatinine >2.5 gm/dl, (12) atherosclerosis within the thoracic aorta, (13) non-contrast CMR angiographic evidence of >30% luminal narrowings in the iliac or femoral arteries, or (14) moderate or severe valvular heart disease.

### Study Design

Participants reported in the morning in the post-prandial state after abstention from all caffeine containing products for at least 12 hours. Heart failure participants also withheld medications after midnight. Each participant underwent knee extensor strength tests, a 4-meter walk speed assessment, and a chair rise test. After these tests, they participated in a maximal exercise test (upright position on an electronically braked bicycle, Medical Graphics, Minneapolis, MN) as previously described in which maximal oxygen consumption and anaerobic threshold was determined by expired gas analysis[5]. During this maximal test, the initial workload began at 12.5 watts for 2 minutes, was advanced to 25 watts for 2 minutes and then by 25-watts increments in 3-minute stages thereafter.

Data from the maximal exercise test were used to identify the level of work that was 30% to 50% of the maximum exercise workload (a modification of the protocol developed by Sullivan, et al. [8,9]); this level of activity established the submaximal, or low level workload for use in the CMR stress test in which aorta and superficial femoral artery LBF would be measured. Twenty-four to 48 hours after maximal testing, participants underwent a non-contrast gradient-echo angiogram of the aorta, iliac and superficial femoral arteries, and a submaximal, supine bicycle exercise test on the CMR table. Before and within 60 seconds of exercise cessation, phase contrast measures of ascending and descending thoracic aorta were accomplished. A recovery period of 5 minutes passed, and then repeat exercise and superficial femoral arterial flow were completed according to previously published techniques within 60 minutes of exercise cessation[10]. This technique was utilized so that the time of image acquisition related to exercise cessation would be similar in the aorta and superficial femoral artery. The exercise during CMR was performed on a nonferromagnetic electronically braked bike (Lode, The Netherlands) beginning at 10 watts for 90 seconds (warm-up), increased to 15 watts for 3 minutes, and finally, increased to the submaximal workload as described.

### CMR of flow in thoracic aorta and superficial femoral artery

CMR was performed with the use of a 1.5-T, GE Twin Speed Scanner (General Electric Medical Systems, Waukesha, WI). Electrocardiographic monitoring leads and a nonferromagnetic brachial blood pressure cuff (In vivo Monitoring Systems, Orlando, FL) were applied for monitoring heart rate and systemic blood pressure throughout the exam. Phase-array surface coils were applied across the chest and around the leg to maximize signal to noise on the images.

**Figure 1:**
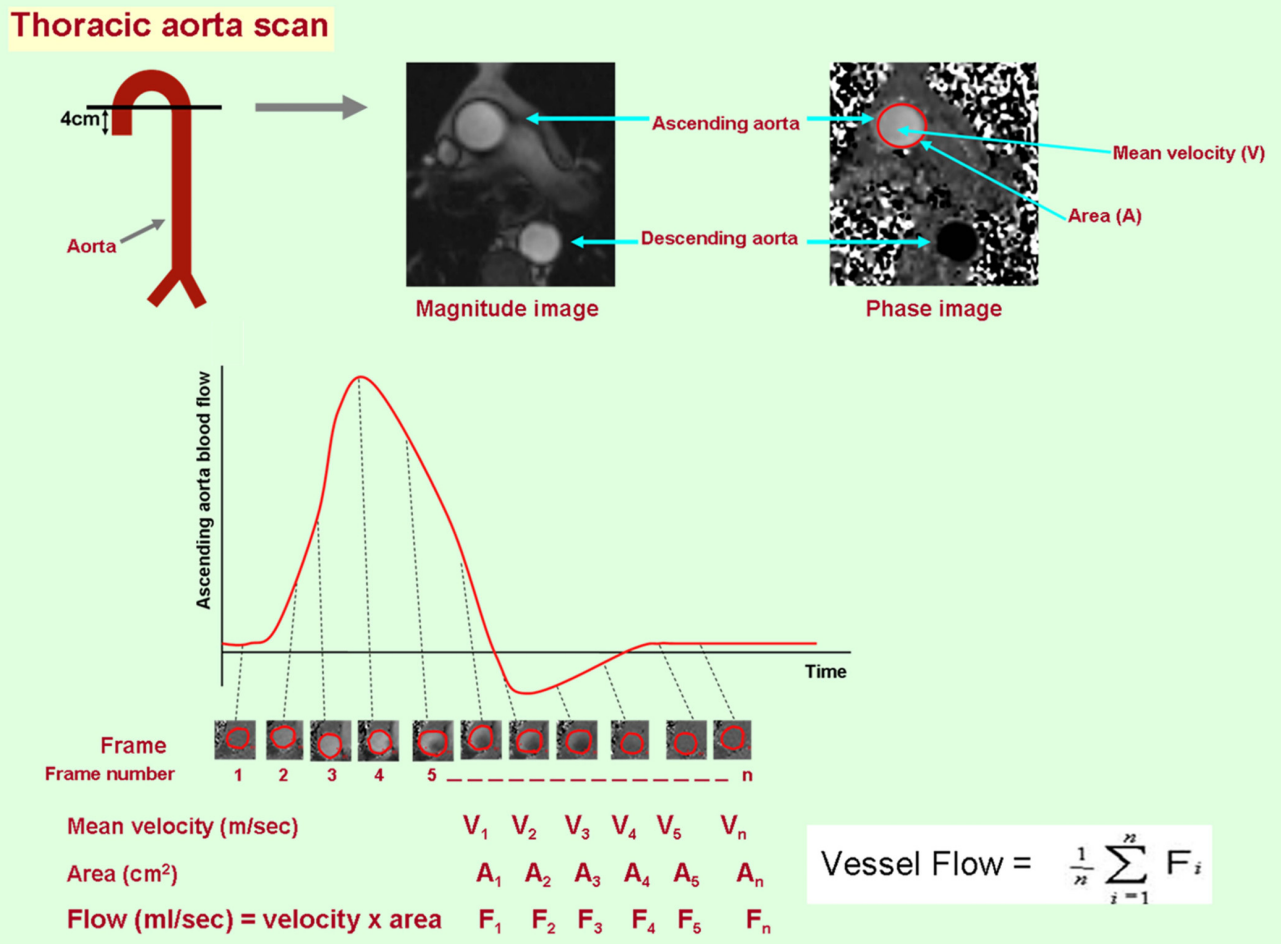
**Methodology used to measure flow in the ascending aorta in one of the study patients**. At the top, a diagram indicating positioning of slices across the ascending aorta 4 cm distal to aortic valve. At these locations, magnitude images and velocity maps were generated with standard phase-contrast techniques. On velocity map, the gray scale intensity for each pixel encodes for velocity in the range of ± m/sec. For each frame of the cardiac cycle, velocity within the vessel is calculated as the average velocity for all the pixels within the lumen. As shown in the lower portion of the figure, flow is determined by summing the flow measurement (Area × mean velocity) the all of the frames acquired across the cardiac cycle.

**Figure 2:**
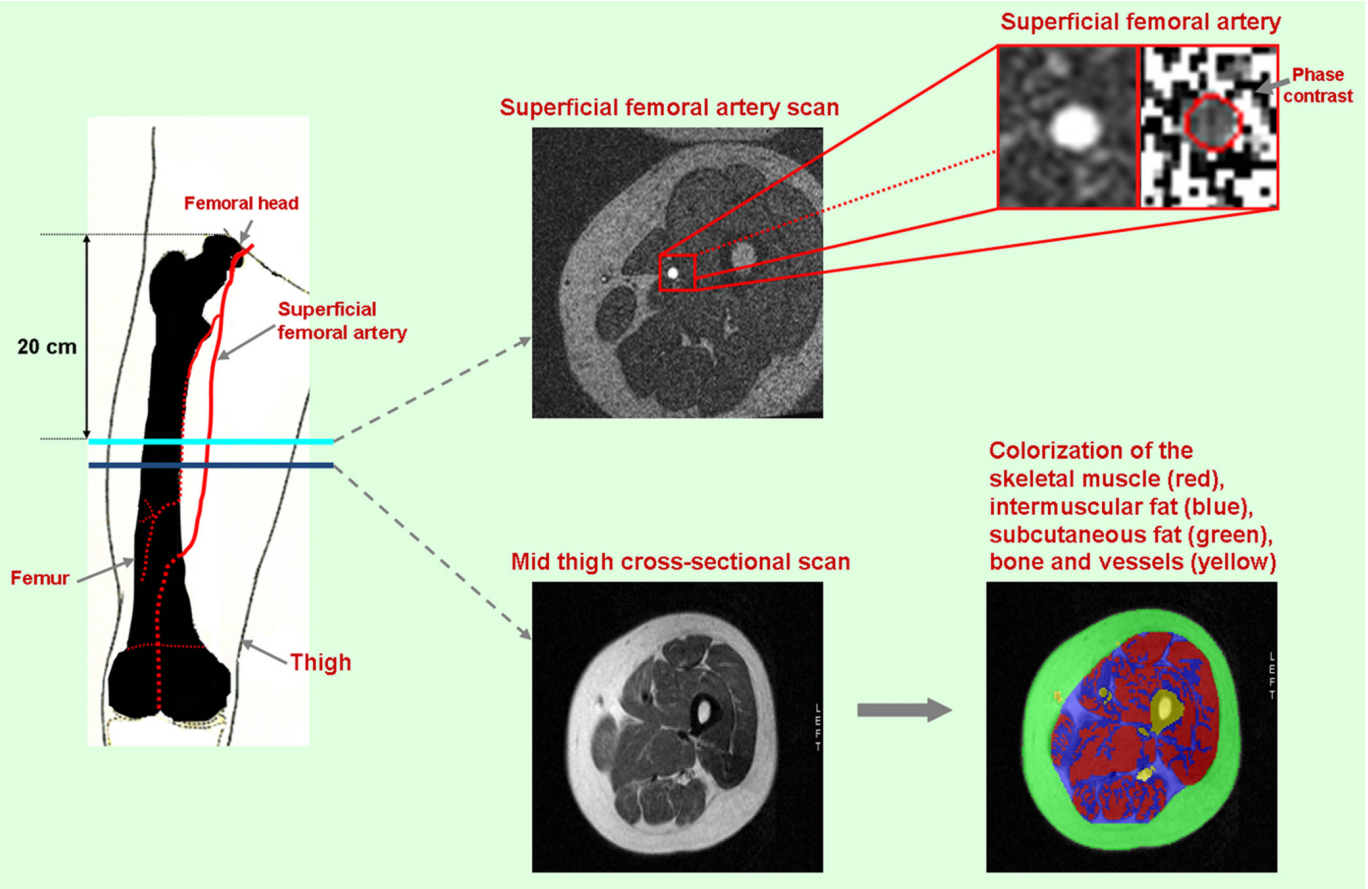
**Methodologies used to acquire superficial femoral artery blood flow and mid-thigh muscle and fat composition**. The location for measurement of flow within the arteries of interest was in the axial plane 20 cm distal to femoral head. The magnified images at the top right demonstrate the cross-section of superficial femoral artery used to measure flow. The images at the bottom demonstrate the mid-thigh cross-sections acquired just distal to the superficial femoral arterial flow images. As shown, color-coded signal thresholding was used to identify fat and skeletal muscle in the middle thigh.

According to previously published techniques,[10] interleaved, phase-contrast gradient-echo sequences were positioned perpendicular to the course of the artery of interest for the purpose of determining cardiac cycle dependent changes in flow. As shown in Figures 1 and 2, the locations for measurement of flow within the arteries of interest were in the axial plane, 4 cm distal to aortic valve level for the ascending thoracic aorta, and 20 cm distal to femoral head for the superficial femoral artery. Measurements in the descending thoracic aorta were obtained from the axial slice, which was used to obtain the ascending aorta flow measurements (Figure 1). Phase-contrast CMR parameters included an 8-mm-thick slice, a 256 × 256 matrix, a 20 cm (for the superficial femoral artery) to 32 cm (for the aorta) field of view (FOV), a 40-degree flip angle, an 13.5-ms repetition time (TR), a 4-ms echo time (TE), and VENC of 150 m/sec. The scan duration was 12 to 15 seconds.

### MR of mid thigh cross-sectional area

Upon completion of the flow sequences, multislice axial images were acquired along the length of the femur. Imaging parameters for these acquisitions included double inversion recovery fast spin-echo images with a 7-mm-thick slice, an echo train length of 32, a TR of 2 × the R-wave to R-wave interval, a 650 msec inversion time, a 42 msec TE, a 20 cm FOV, and a 256 × 256 matrix (pixel sizes = 0.78 mm × 0.78 mm). Using the Tomo-Slice software as previously described, the areas of skeletal muscle, adipose, and other (bone, interstitial, vascular) tissue were determined (Figure 2)[11].

All CMR data were analyzed by individuals blinded to patient condition (HF versus healthy), individual components of the CMR exam (thigh muscle area versus flow), or the measures of physical function or exercise capacity. Also, individuals reporting data on physical function exercise capacity were blinded to the results of CMR analyses.

### Statistical analysis

Baseline population characteristics of healthy and HF groups were compared using Student's t-test (for continuous variables) or the Chi-square test (for categorical variables). Distributions of the outcome measurements (physical performance, exercise capacity, mid thigh composition and arterial blood flows) were examined, and appropriate transformations were made if necessary. Since superficial femoral LBF is affected by height, BSA, and mid-thigh cross-sectional muscle area, three adjusted versions of superficial femoral LBF were obtained by dividing each factor. Student's t-tests were used to compare the 2 groups (HF and healthy) on these outcomes. In order to adjust for other confounding factors such as age, gender, and LVEF, we also fitted two separate analysis of covariance models to compare superficial femoral arterial blood flow measurements (rest, stress and the difference between rest and stress) between the 2 groups after adjusting for age, gender, or body surface area, height, LVEF, and thigh muscle area, respectively. All tests were two-sided with significance levels of 0.05. All analyses were performed with SAS 9.1 (Cary, NC). All authors had full access to the data and take full responsibility for the integrity of the data, and have read and agree to the manuscript as written.

All authors had full access to the data and take full responsibility for the integrity of the data, and have read and agree to the manuscript as written.

## Results

All participants completed the study protocol and their demographic data are displayed in Table 1. Participants with HF displayed a higher incidence of hypertension (67%) and remote myocardial infarction (33%), compared to the healthy older individuals. Elderly HF participants trended toward reduced knee extensor strength (maximum torque 94.5 ± 58.6 vs. 124.5 ± 45.1 Nm; *p *= 0.18) and delayed chair raise (11.7 ± 2.2 vs. 13.5 ± 1.9 m/sec, *p *= 0.06), respectively, compared to healthy older individuals. Four-meter walk speed (0.98 ± 0.15 vs. 1.35 ± 0.24 m/sec, *p *< 0.001) was reduced in elderly HF participants relative to the healthy older individuals, respectively. Heart failure participants demonstrated reduced peak exercise capacity (peak VO_2_, *p *= 0.01) compared to healthy subjects (Table 2).

**Table 1:** Demographic data (mean ± standard deviation)

	Healthy (n = 13)	Heart Failure (n = 12)	p-value
**Male, n(%)**	6(46%)	6(50%)	0.85
**Age (years)**	67 ± 5	70 ± 8	0.29
**Weight (kg)**	76 ± 11	81 ± 14	0.41
**Height (cm)**	171 ± 10	169 ± 10	0.5
**BSA (m^2^)**	1.9 ± 0.2	1.9 ± 0.2	0.64
**LVEF(%)**	66 ± 7	48 ± 25	0.04*
**Diabetes, n(%)**	0(0%)	1(8%)	0.48
**Hypertension, n (%)**	0(0%)	8(67%)	0.001*
**Hypercholesterolemia**	0(0%)	8(67%)	0.001*
**Prior myocardial infarction**	0(0%)	4(33%)	0.04*
**COPD/Asthma**	0(0%)	2(17%)	0.22
**Medication, n(%)**			
Betablocker	0(0%)	8(67%)	<0.001*
ACE inhibitor	0(0%)	6(50%)	0.01*
ARB	0(0%)	2(17%)	0.22
Calcium channel blocker	1(8%)	3(25%)	0.32
Digoxin	0(0%)	2(17%)	0.22
Hydralazine	0(0%)	1(8%)	0.48
Nitrate	0(0%)	2(17%)	0.22
Aspirin/NSAID	9(69%)	6(50%)	0.43
Diuretic	0(0%)	6(50%)	0.01*
Statin	0(0%)	5(42%)	0.01*
Calcium supplement	5(38%)	2(17%)	0.38
Prior hormone replacement therapy	1(8%)	0(0%)	1
**Hemoglobin (g/dl)**	14.9 ± 1.7	13.1 ± 1.4	0.02*
**BNP**	20 ± 12	118 ± 132	0.06
**Serum sodium level**	143 ± 2	142 ± 2	0.61
* = p < 0.05			

Abbreviation: ACE = Angiotensin converting enzyme; ARB = Angiotensin II receptor blocker; BMI = Body mass index; BNP = B-Type natriuretic peptide; BSA = Body surface area; BUN = Blood urea nitrogen; COPD = chronic obstructive airway disease; FBS = Fasting blood sugar; LVEF = left ventricular ejection fraction; NSAID = Non-steroid anti-inflammatory drug

**Table 2:** Maximal (Peak) upright bicycle exercise and gas exchange analysis

	Healthy	Heart Failure	p-value
	mean ± SD	mean ± SD	
Peak work load (watts)	97.8 ± 19.1	82.7 ± 22.3	0.002
Peak VO2 (ml/kg/min)	19.8 ± 5.7	13.9 ± 3.2	0.01
Max RER	1.03 ± 0.33	1.04 ± 0.07	0.95
Peak Heart rate	138 ± 28	111 ± 20	0.01
Peak Systolic blood pressure	195 ± 24	156 ± 30	0.01
Peak Diastolic blood pressure	81 ± 9	78 ± 8	0.51
Peak RPP	28295 ± 7412	17842 ± 5616	0.003

Abbreviations: PMHR = percent maximum heart rate (adjusted for age); RER = Respiratory exchange ratio; RPP = rate pressure project; VO2 = oxygen uptake

**Figure 3:**
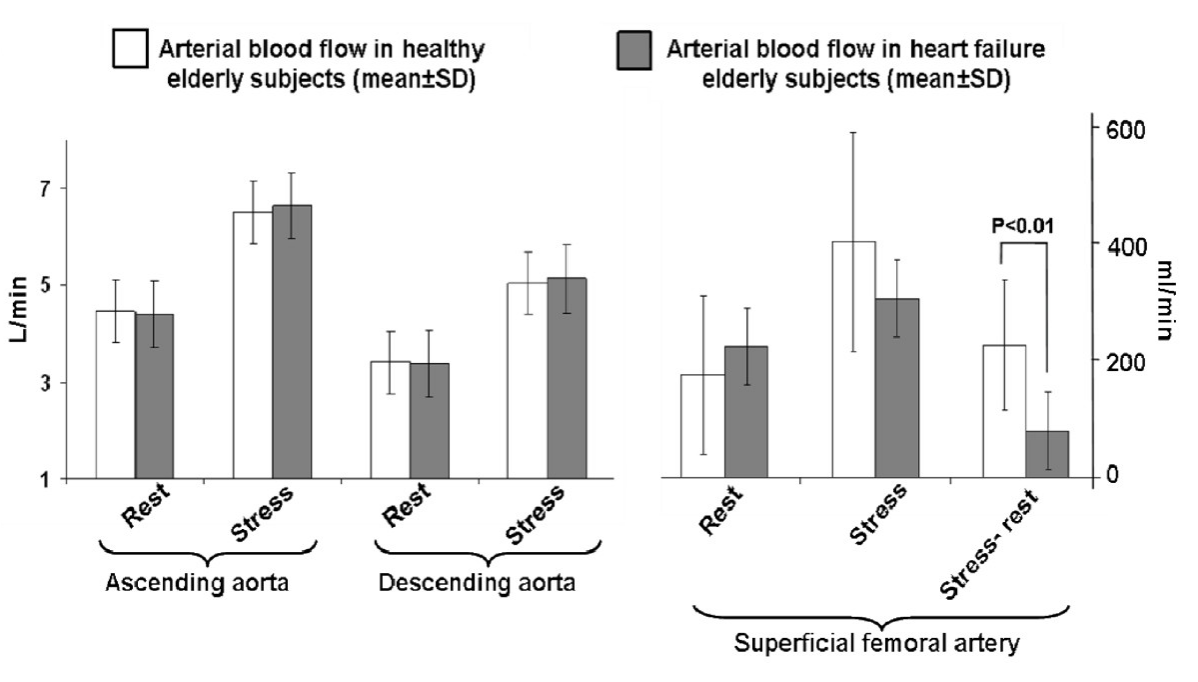
**Bar graphs demonstrating rest and submaximal exercise induced measures of arterial blood flow (mean ± standard deviation) in the ascending aorta, descending aorta, and the superficial femoral artery**. White bars indicate data from the healthy elderly participants, and gray bars indicate data from the heart failure (HF) elderly participants. As shown, although blood flow in the aorta (ascending and descending portions) augmented with submaximal exercise in both HF and healthy elderly participants, blood flow did not display a commensurate increase in the superficial femoral artery in participants with HF.

After submaximal exercise, the change in superficial femoral artery LBF was lower in HF participants (79 ± 92 ml/min) compared to healthy participants (222 ± 108 ml/min; *p *= 0.002). This occurred even though resting, stress induced, and the stress-rest difference in the descending aortic flow were similar between the 2 groups (Figure 3). Peak VO_2 _was correlated with peak superficial femoral arterial flow after submaximal exercise (r = 0.67; p = 0.003). The watts achieved during CMR exercise were correlated with peak superficial femoral artery blood flow (r = 0.51; p = 0.01), but not with the stress-rest difference in superficial femoral artery flow (r = -0.34; p = 0.11).

To determine if the presence of prior myocardial infarction, diabetes, hypertension, or hypercholesterolemia could have accounted for the differences in flow observed in the superficial femoral artery of our HF and healthy participants, we adjusted our results for the presence of these factors. After these adjustments, submaximal exercise induced difference (stress-rest) in superficial femoral artery LBF persisted between our healthy and HF groups (difference significant at *p *≤ 0.05 after including all clinical factors). Also, the similarity between measures of ascending and descending aorta blood flow persisted between HF and healthy elderly participants (*p *> 0.2 after considering all clinical factors). Since body habitus and particularly thigh muscle size or mass can be different in participants with HF relative to normals, we performed analyses that accounted for body habitus and thigh muscle area. Images of the middle thigh demonstrated trends toward reductions in thigh muscle area and elevations in subcutaneous fat and intermuscular fat in the elderly HF participants versus the healthy participants (0.1<*p *≤ 0.15 for all areas assessed thigh components). After adjusting our blood flow measurements for age, gender, body surface area, and thigh muscle area, the differences in stress-rest measures of superficial femoral arterial LBF persisted (*p *= 0.002 to 0.003 for all).

The 13 healthy elderly participants exhibited a LVEF of 66 ± 7%, which was on average higher than the participants with HF (average LVEF of 48 ± 25%). As has been previously shown in elderly individuals with HF,[12] the LVEF in our HF participants ranged from severely reduced to preserved (15% to 86%). These findings are commensuruate with the syndrome of heart failure and a normal LVEF (HFNEF), heart failure and a reduced LVEF (HFREF), and a mixture of the two. With this range of LVEF in our study, we were able to perform analyses that evaluated the potential influence of LVEF on our results. Importantly, after adjusting our stress-rest changes in superficial femoral artery LBF for resting LVEF, the differences between our HF and healthy elderly groups persisted (*p *= 0.03) indicating that the difference observed in superficial femoral arterial flow was present across the range of LVEF studied.

## Discussion

Older patients with HF have particularly severe exercise intolerance, the primary symptom of chronic HF[12,13]. Studies in younger patients with HF have suggested that reduced LBF can contribute to exercise intolerance[14,15]. In the present study, we studied older patients with HF and compared them to an age-matched group of healthy elderly subjects. The characteristics of the HF participants in this study were representative of prior populations of elderly HF[12,16,17]. Using expired gas analysis during exhaustive upright exercise, we found that elderly HF participants exhibited severely decreased exercise capacity (Table 2), which is consistent with previous reports from our group and others[12,18,19]. All participants (HF and healthy) in this study were carefully screened to exclude factors known to alter arterial LBF, including evidence of peripheral arterial atherosclerosis.

Using phase contrast CMR to measure flow in the superficial femoral artery, and the ascending and descending aorta at rest and immediately after supine submaximal exercise, we found that older patients with HF have significantly reduced LBF despite similar responses in cardiac output (Figure 3). This phenomenon persisted after accounting for a wide variety of potentially confounding factors, including thigh muscle area, LVEF, gender, body size, and age. This finding occurred even though HF participants received medical therapy with angiotensin converting enzyme inhibitors or receptor blockers that could have improved blood flow (Table 3). These data suggest that older HF patients have abnormal distribution of blood flow to the peripheral vascular system even though cardiac output is preserved, and that reduced LBF may be an important contributor to their severe exercise intolerance.

**Table 3:** Hemodynamic data during supine submaximal bicycle and blood flow measurement

	Healthy mean ± SD	Heart Failure mean ± SD	p-value
**Resting**			
*Heart rate*	65 ± 10	62 ± 7	0.51
Systolic blood pressure	105 ± 48	111 ± 56	0.75
Diastolic blood pressure	55 ± 27	53 ± 27	0.87
RPP	6942 ± 974	6805 ± 1023	0.92
**Stress (30% to 50% of maximal workload)**			
*Heart rate*	86 ± 16	85 ± 12	0.89
Systolic blood pressure	123 ± 57	131 ± 63	0.76
Diastolic blood pressure	59 ± 31	63 ± 31	0.74
RPP	11219 ± 1466	11555 ± 1696	0.88
Watts	37 ± 5	26 ± 15	0.001
Watts as % of Peak Workload	38% ± 9	41% ± 9	0.52
**Ascending aorta blood flow (ml/min)**			
*Unadjusted*			
Rest	4473 ± 259	4407 ± 447	0.9
Stress	6508 ± 383	6638 ± 549	0.85
Difference between rest and stress	2035 ± 324	2231 ± 31	0.67
*Adjusted for height*			
Rest	26 ± 6	26.03 ± 8.74	0.96
Stress	38 ± 8	39 ± 11	0.74
Difference between rest and stress	12 ± 7	13 ± 6	0.59
**Descending aorta blood flow (ml/min)**			
*Unadjusted*			
Rest	3404 ± 714	3383 ± 1021	0.95
Stress	5048 ± 1189	5132 ± 1293	0.87
Difference between rest and stress	1644 ± 812	1749 ± 812	0.75
*Adjusted for height*			
Rest	20 ± 4	20 ± 6	0.95
Stress	29 ± 6	30 ± 7	0.71
Difference between rest and stress	10 ± 5	10 ± 5	0.64
**Superficial femoral arterial blood flow**			
***Unadjusted***			
Rest (ml/min)	173 ± 133	221 ± 184	0.46
Stress (ml/min)	394 ± 184	300 ± 180	0.21
Difference between rest and stress (ml/min)	222 ± 108	79 ± 92	0.002**
***Adjusted for height***			
Rest (ml/min/cm)	1.0 ± 0.7	1.3 ± 1.0	0.41
Stress (ml/min/cm)	2.3 ± 0.9	1.8 ± 1.0	0.21
Difference between rest and stress (ml/min/cm)	1.3 ± 0.6	0.5 ± 0.6	0.002**
***Adjusted for BSA***			
Rest (ml/min/m^2^)	89.8 ± 63.7	108.4 ± 83.7	0.54
Stress (ml/min/m^2^)	206.0 ± 89.6	150.3 ± 81.6	0.12
Difference between rest and stress (ml/min/m^2^)	116.1 ± 56.1	41.9 ± 49.0	0.002**
***Adjusted for mid thigh cross-sectional muscle area***			
Rest (ml/min/cm^2^)	2.0 ± 1.3	2.6 ± 2	0.39
Stress (ml/min/cm^2^)	4.6 ± 1.9	3.7 ± 2.1	0.28
Difference between rest and stress (ml/min/cm^2^)	2.6 ± 1.2	1.1 ± 1.4	0.01**
***Adjusted for age and gender***			
Rest (ml/min)	181.8 ± 41.7†	216.1 ± 43.4†	0.58
Stress (ml/min)	403.9 ± 48.0†	296.0 ± 50.0†	0.14
Difference between rest and stress (ml/min)	222.0 ± 29.5†	79.9 ± 30.7†	0.003**
***Adjusted for LVEF***			
Rest (ml/min)	214.8 ± 54.3†	178.0 ± 51.5†	0.65
Stress (ml/min)	433.9 ± 65.0†	267.0 ± 62.3†	0.10
Difference between rest and stress (ml/min)	219.1 ± 37.1†	88.9 ± 35.2†	0.03**

† = mean ± standard error, ** = p < 0.03

Abbreviations: BSA = body surface area; LVEF = left ventricular ejection fraction; PMHR = peak maximum heart rate; RPP = rate pressure project

The findings of the present study are supported by those of prior studies, which have demonstrated that LBF is reduced during exercise in younger and middle aged patients with exercise intolerance,[14,15] even though submaximal cardiac output increases. The present study increases our understanding of the relationship between LBF and exercise capacity by extending these observations to older patients who comprise the majority of the population with HF, and who often have complex etiologies of HF with more severe exercise intolerance compared to younger patients[12]. Lower extremity blood flow has been shown to decrease with normal aging[15,20]. Importantly, in this study using highly precise measures of blood flow and well characterized participants, differences in LBF between our HF and healthy elderly persist after accounting for the clinical conditions (hypertension, hypercholesterolemia, and diabetes) known to effect cardiac output and blood flow in the elderly.

The normal physiological response to exercise involves the control of LBF. With the onset of exercise, selective vasoconstriction temporarily decreases blood flow to splanchnic and other non-critical arterial beds[21,22]. In combination with selective vasodilatation of the femoral and distal leg arteries, this allows efficient distribution of cardiac output to the exercising leg muscles in order to facilitate physical work[23,24]. It is known that normal aging is associated with significant alterations in peripheral arterial blood flow responses at rest and after a variety of stressors, including exercise [21-24]. Thus, inclusion of an age-matched healthy control group was an important design feature of the present study. Our results indicate that older HF patients have reduced leg blood flow with exercise beyond that which is associated with normal aging.

Although we did not measure blood flow to the viscera in the present study, several studies have demonstrated in humans that blood volume decreases in splanchnic, renal, and superior mesenteric arteries during or immediately after exercise[25,26] Osada, et al., [27] has shown in healthy subjects that there is redistribution of blood flow away from the abdominal viscera, even during low-intensity submaximal exercise, and that this reduction is proportional to an increase in oxygen consumption. Others have shown that non-leg blood flow to non-exercising regions (i.e. visceral organs and fat), [28,29] and exercising organs (i.e. trunk and respiratory muscles)[30] during exercise may contribute to leg muscle hypoperfusion and exercise intolerance. We recognize that we have no data regarding distribution of abdominal blood flow in our current study. However, our results are important in that we confirm that blood flow entering the abdomen during submaximal exercise is equivalent in elderly with or without HF. Our results suggest that mechanisms which redistribute abdominal flow during exercise may be inoperative in older HF patients and may account for reduced LBF during submaximal exercise (the level of exercise for which activities of daily living are performed).

Although the control of LBF in the elderly is not completely understood, several studies have implicated increased sympathetic nerve activity, vascular smooth muscle cell intimal medial thickening, endothelial dysfunction, and chronic cytokine activation as mechanisms which alter redistribution of blood flow during exercise[23,31]. We have previously shown that older HF patients have reduced aortic distensibility which correlates with exercise intolerance,[18,32] and that older HF patients with a reduced LVEF have impaired flow-mediated arterial dilation (FMD), indicative of endothelial dysfunction[16]. In this study we did not measure FMD in the subjects. The HF subjects demonstrate a similar increase in brachial artery blood pressure with exercise as control subjects. Despite this, the relative increase in LBF was blunted. Recognizing that mean arterial pressure in the brachial artery is indeed representative of central aortic mean arterial pressure (especially in older subjects where significant atheroslcerosis was an exclusion), these data suggest that a potential mechanism for the relatively blunted increase in LBF in the HF subjects is an impaired active hyperemia and/or a relative inability of the HF subjects to drop their systemic vascular resistance to exercise[33].

An important consideration regarding our results pertains to the fact that alterations in the control of LBF are modifiable. Beere et al.,[34] have shown that exercise conditioning partially reverses the normal age-related reduction in LBF during exercise, resulting in improved exercise capacity. In HF patients, exercise training and angiotensin converting enzyme inhibition improve exercise induced LBF and endothelial dysfunction, which leads to improvements in exercise intolerance[15,35-37].

Since the LVEF was lower in our HF group relative to our healthy older participants, due to inclusion of participants with HFREF in addition to those with HFNEF, we suspected that cardiac output would be reduced with submaximal exercise. This did not occur. Although the LVEF of the HF group was lower than that observed in the healthy group, the lower submaximal exercise induced difference (stress-rest) in superficial femoral arterial blood in the HF participants persisted after adjustment for LVEF (p = 0.03). Our data indicate that the LVEF per se and the impact of LVEF on cardiac output, are not responsible for the reduced submaximal exercise induced superficial femoral arterial flow observed in our HF participants. Our data does not allow us to conclude whether other indirect consequences of a poor LVEF, or the differential influence in factors, such as autonomic dysfunction or heightened inflammatory cytokines, which may be disproportionately present in individuals with HFNEF versus HFREF, may have been responsible for the reduction in submaximal exercise induced superficial femoral LBF observed in our HF subjects.

This study has potential limitations. First, we did not perform our CMR blood flow measurements during maximal exercise; thus we cannot prove that reduced LBF was the sole cause of the severely reduced exercise capacity of the older HF patients. Second, we did not set an absolute workload for our participants; this resulted in the HF participants performing a lower absolute measure of work compared to controls. Absolute determinations could have been highly variable (25% to near 100%) of maximum work for our HF participants dependant on their level of conditioning. We particularly wanted to avoid having HF participants come close to their anaerobic thresholds, a point during exercise when physiology changes, during our exercise protocol. Instead, we used a relative workload value based on all participants exhibiting maximum efforts (respiratory exchange ratios > 1.02), and then taking 30%-50% of this level to ascribe the work needed to perform submaximal exercise during our protocol. This has the effect of producing comparable levels of submaximal work across all of the study participants.

Third, we cannot definitively exclude subclinical atherosclerosis as a potential contributor to the reduced LBF in the superficial femoral artery. We vigorously screened all participants for atherosclerosis in the large arteries (carotid, femoral, iliac, and thoracic aorta) with ultrasound and CMR. We did not perform brachial/ankle pressure assessments which can screen for peripheral arteriosclerosis. Importantly, the intergroup differences in leg blood flow persisted after adjusting for risk factors for atherosclerosis, including hypercholesterolemia, hypertension, diabetes, and prior myocardial infarction. Fourth, most of our participants were Caucasian; thus, we have little data on those of other race.

Fifth, the sample size in our study was not large. However, we were able to draw important conclusions from our study population due to the high precision of our CMR measurements. Earlier studies of LBF required invasive techniques and dual catheters to measure leg blood flow and cardiac output. The present study included a noninvasive CMR method for measuring cardiac output and LBF changes with submaximal exercise. This methodology is based upon CMR measures of cardiac output and blood flow, which we and others have previously shown to be accurate (intraobserver and interobserver coefficient of variation for measuring volume was 1.2% and 1.3%),[38] highly reproducible (intrasubject coefficient of variation for femoral artery flow volume was 16%),[38-42] and well-suited for performing serial evaluations to determine LBF in the elderly.

Finally, both a strength and a weakness of the current study was that the HF subjects held their HF medications the night before the study. This was a strength in that the potentially confounding effects of these medications were removed, thus allowing the study of 'native' cardiac and vascular function. A potential weakness of this strategy is that HF patients are prescribed these therapies to improve survival; thus they frequently take them. It is conceivable that the magnitude of the LBF differential may have been less, if for example, an ACEI or ARB had been continued in the HF participants at the time of exercise CMR.

In conclusion, in elderly patients with HF, functional impairment, and exercise intolerance, submaximal exercise induced femoral arterial blood flow is reduced even though cardiac output is preserved. This finding occurs after accounting for thigh muscle area, age, gender, and co-morbidities associated with poor vascular function. In contrast to our original hypothesis, these results indicate that mechanisms other than low cardiac output are responsible for reduced LBF during submaximal exercise in the elderly with HF. For these reasons, future studies should address whether a) inappropriate distribution of LBF in the absence of abnormalities of submaximal exercise induced changes in cardiac output are present in elderly disabled individuals with exercise intolerance, and b) therapies that improve LBF without necessarily modifying LVEF can reduce exercise intolerance and disability observed in elderly HF.

## Competing interests

Drs. Hundley and Hamilton have an ownership stake in MRI Cardiac Services, Inc. that creates software in MRI image management and display.

## Authors' contributions

CP acquired, analyzed, and interpreted the data, and drafted the manuscript. DWK participated in its design and coordination, handled funding and supervision, acquired, analyzed, and interpreted the data, and helped to draft the manuscript. SBK handled funding and supervision. CAH aided in acquiring, analyzing, and interpreting the data. BN aided in acquiring, analyzing, and interpreting the data. XI performed the statistical analysis. PHB aided in acquiring, analyzing, and interpreting the data. WGH conceived of the study, participated in its design and coordination, handled funding and supervision, acquired, analyzed, and interpreted the data, and helped to draft the manuscript. All authors made critical revision of the manuscript for important intellectual content and approved of the final manuscript.
